# The B Subunit of PirAB*^vp^* Toxin Secreted from *Vibrio parahaemolyticus* Causing AHPND Is an Amino Sugar Specific Lectin

**DOI:** 10.3390/pathogens9030182

**Published:** 2020-03-03

**Authors:** Marcelo Victorio-De Los Santos, Norberto Vibanco-Pérez, Sonia Soto-Rodriguez, Ali Pereyra, Edgar Zenteno, Patricia Cano-Sánchez

**Affiliations:** 1 Laboratorio de Bacteriología. Centro de Investigación en Alimentación y Desarrollo, A.C. Unidad de Acuacultura y Manejo Ambiental, Av. Sábalo-Cerritos S/N A.P. 711, Mazatlán, Sinaloa 82112, Mexico; 2 Laboratorio de Investigación en Biología Molecular e Inmunología, Unidad Académica de Ciencias Químico Biológicas y Farmacéuticas, Universidad Autónoma de Nayarit, Ciudad de la Cultura, Tepic, Nayarit 63190, Mexico; 3 Departamento de Bioquímica, Facultad de Medicina, Universidad Nacional Autónoma de México, Circuito Exterior, Ciudad Universitaria, Coyoacan, CDMX 04510, Mexico; ali@bq.unam.mx (A.P.); ezenteno@unam.mx (E.Z.); 4 Laboratorio de Biología Molecular, Instituto de Química, Universidad Nacional Autónoma de México, Circuito Exterior, Ciudad Universitaria, Coyoacan, CDMX 04510, Mexico; pcano@iquimica.unam.mx

**Keywords:** PirAB*^vp^* toxin, lectin, binary toxin, AHPND, *Vibrio parahaemolyticus*, amino-sugars, glycosaminoglycans

## Abstract

*Vibrio parahaemolyticus* (*Vp*) is the etiological agent of the acute hepatopancreatic necrosis disease (AHPND) in *Penaeus vannamei* shrimp. *Vp* possesses a 63–70 kb conjugative plasmid that encodes the binary toxin PirA*^vp^*/PirB*^vp^*. The 250 kDa PirAB*^vp^* complex was purified by affinity chromatography with galactose-sepharose 4B and on a stroma from glutaraldehyde-fixed rat erythrocytes column, as a heterotetramer of PirA*^vp^* and PirB*^vp^* subunits. In addition, recombinant pirB (rPirB*^vp^*) and pirA (rPirA*^vp^*) were obtained. The homogeneity of the purified protein was determined by SDS-PAGE analysis, and the yield of protein was 488 ng/100 μg of total protein of extracellular products. The PirAB*^vp^* complex and the rPirB*^vp^* showed hemagglutinating activity toward rat erythrocytes. The rPirA*^vp^* showed no hemagglutinating capacity toward the animal red cells tested. Among different mono and disaccharides tested, only GalNH_2_ and GlcNH_2_ were able to inhibit hemagglutination of the PirAB*^vp^* complex and the rPirB*^vp^*. Glycoproteins showed inhibitory specificity, and fetuin was the glycoprotein that showed the highest inhibition. Other glycoproteins, such as mucin, and glycosaminoglycans, such as heparin, also inhibited the activity. Desialylation of erythrocytes enhanced the hemagglutinating activity. This confirms that Gal or Gal (β1,4) GlcNAc are the main ligands for PirAB*^vp^*. The agglutinating activity of the PirAB*^vp^* complex and the rPirB*^vp^* is not dependent on cations, because addition of Mg^2+^ or Ca^2+^ showed no effect on the protein capacity. Our results strongly suggest that the PirB*^vp^* subunit is a lectin, which is part of the PirA/PirB*^vp^* complex, and it seems to participate in bacterial pathogenicity.

## 1. Introduction

The worldwide production of *Penaeus vannamei* shrimp has suffered significant losses in Asia [1,2] and Latin America [3,4]. This has been due to the acute hepatopancreatic necrosis disease (AHPND), which is considered an emerging shrimp disease. Specific strains of *Vibrio parahaemolyticus* (*Vp*) that were first reported in Mexico in 2013 are the etiological agents of the disease. The *Vp* strains harbor a 63–70 kb conjugative plasmid [5]. The plasmid called pVA1 encodes a binary PirA/PirB toxin homologous to the *Photorhabdus luminiscens* insect-related (Pir) binary toxin [5,6,7,8]. Recombinant PirA*^vp^* and PirB*^vp^* were reported to be the primary virulence factor that causes massive sloughing of the tubule epithelial cells of the shrimp hepatopancreas (Hp), causing 100% cumulative mortality in 24 h post-infection [8].

The PirAB*^vp^* binary toxin has been identified in other *Vibrio* species belonging to the *Harveyi* clade, such as *V. harveyi* [9], *V. campbellii*, and *V. owensii* [5,10,11]. The presence of genes encoding PirA and PirB was reported in the Gram-positive bacterial species *Micrococcus luteus* [12]. This bacterium was isolated in 2006 from the Hp of farmed *P. vannamei* shrimp collected in Nayarit, Mexico; it is possible that the toxin was acquired by horizontal gene transfer from AHPND isolates [13,14]. The genome of *Vibrio punensis*, belonging to the *Orientalis* clade, also contains the toxigenic genes that cause AHPND in shrimp [15]. The presence of these genes in different bacterial species is a potential risk for the spread of emerging diseases. The clinical signs of shrimp affected with AHPND are a pale Hp, empty gut, anorexia, and lethargy accompanied by pathognomonic lesions: massive sloughing of tubule epithelial cells of the shrimp Hp [6,16]. Most authors suggest that the PirAB*^vp^* toxin acts as a pore-forming toxin that kills the hepatopancreatic cells of shrimp, although this has not been experimentally demonstrated. Moreover, the specific mechanisms used to recognize specific cellular receptors for the PirAB*^vp^* toxin have not yet been elucidated [6,16,17]. 

AB toxins are synthesized by a variety of bacteria, pathogens, and plants. The most well-known AB toxins include the cholera toxin, shiga toxin, pertussis toxin, anthrax, and ricin [18]. Many of these AB toxins contain two functional regions: an enzymatically active cytotoxic subunit and a region that recognizes cell surface receptors [19]. Ricin is a type of AB toxin with lectin activity. It is isolated from the castor tree (*Ricinus cummunis*), one of the most poisonous plants in the world. Lectins are a group of proteins that share the ability to recognize and bind to specific carbohydrate structures. They are ubiquitous in nature. They are present in various types of organisms, and their particular interaction with carbohydrate structures plays a role in different biological processes, including the control of pathogens and predators [20,21].

Currently, there are no reports of AB toxins with lectin activity in marine bacteria, so the isolation and determination of the specificity of both subunits for sugars are necessary to identify adhesion molecules or receptors on the hepatopancreatic epithelial cells. This work aims to identify the specificity of the toxin from sugars of the subunits of the PirAB*^vp^* toxin secreted by *Vibrio parahaemolyticus*, particularly PirB*^vp^*.

## 2. Materials and Methods

### 2.1. Growth Conditions of E. coli Strains and Vibrio Parahaemolyticus 

*Escherichia coli* BL21 CodonPlus-RIL (Agilent Technologies, Inc., Santa Clara, CA, USA) and *E. coli* Top10 (Invitrogen, Carlsbad, CA, USA) were used for the transformation and replication, respectively, of the genes coding the toxins PirA*^vp^* and PirB*^vp^* and cloned into the pET system plasmid. The *E. coli* culture was maintained on Luria–Bertani (LB) agar plates supplemented with the appropriate antibiotics. For rPirA*^vp^*, kanamycin (50 μg/mL) and chloramphenicol (34 μg/mL) were used; for rPirB*^vp^*, ampicillin (100 μg/mL) and chloramphenicol (34 μg/mL) were used. For protein expression, cultures were grown in LB at 37 °C and induced at 30 °C with shaking (200 rpm). 

The highly virulent M0904 strain of *Vibrio parahaemolyticus* (*Vp*), which causes AHPND in shrimp, was used. It was isolated from shrimp hepatopancreas [16]. A pure culture of *Vp*M0904 was obtained by inoculation in tryptic soy broth (TSB, Bioxon, Mexico), according to Soto-Rodriguez et al. [16].

### 2.2. Cloning and Expression of Recombinant PirA^vp^ and PirB^vp^

The recombinant proteins rPirA*^vp^* and rPirB*^vp^* were cloned separately, transformed using the *E. coli* BL21 CodonPlus-RIL cell line, and induced with isopropyl-β-D-1-thiogalactopyranoside (IPTG). Briefly, the PirA*^vp^* sequence was purified using the resulting amplicon for a PCR with primers containing NdeI and HindIII (Thermo-Fisher Co., San José, CA, USA) restriction sites: PirA-NdeI-Forward: 5′-AAT CAT ATG AGT AAC AAT ATA AAA CAT G-3′ and PirA-HindIII-Reverse: 5′-AAT AAG CTT AGT GGT AAT AGA TTG TAC AG-3′. The PCR products were purified and double digested with NdeI and HindIII enzymes before ligation into the vector pET-28a (Promega, Co., Madison, WI, USA) for the transformation of *E. coli* BL21 CodonPlus-RIL. Protein expression was induced with 0.5 mM of IPTG at 30 °C for 16 h; later, the cells were collected by centrifugation and stored at −20 °C until the purification process. A similar protocol was followed for the rPirB*^vp^* expression. The PCR product was obtained using primers containing NcoI and XhoI restriction sites. The primers were PirB-NcoI (Forward-AAT CCA TGG GTA CTA ACG AAT ACG TTG TAA C-3′) and PirB-XhoI (Reverse-TAA CTC GAG TTA CTA CTT TTC TGT ACC AAA TTC AT-3′). The PCR product was purified, double digested with NcoI and XhoI (Thermo-Fisher), and ligated into the plasmid pET32c (Promega) to transform *E. coli* BL21 CodonPlus-RIL for the expression of a thioredoxin (TRX) fusion protein and His6 tag. Recombinant protein expression was induced by 0.5 mM IPTG at 30 °C for four h; then, it was separated by centrifugation and stored at −20 °C until the purification process. 

### 2.3. Purification of rPirA^vp^ and rPirB^vp^ Toxins

Purification of the recombinant proteins was carried out by one-step Ni-affinity chromatography on a Ni-Sepharose Fast Flow resin (GE Healthcare, Chicago, IL, USA). Briefly, bacterial cells were washed using buffer A (50 mM Tris-HCl, 300 mM NaCl, 10 mM imidazole, 5% glycerol, 0.25 mM DDT, pH 8.0) and the cell suspension was sonicated at 42 W on ice for 5 min (5 s on, 15 s off). Cell debris was removed by centrifugation at 24,000× *g* for 45 min at 4 °C. After sonication, the supernatant solution was loaded on a Ni-affinity column, which had already been equilibrated with buffer A. To remove any nonspecifically bound proteins, the column was extensively washed with buffer B (50 mM Tris-HCl, 300 mM NaCl, 20 mM imidazole, 5% glycerol, 0.25 mM DDT, pH 8.0). For rPirA*^vp^*, the protein was eluted with buffer C (50 mM Tris–HCl, 300 mM NaCl, 5% glycerol, 0.25 mM DTT, pH 8.0) with different concentrations of imidazole (20, 50, 100, 300, and 500 mM) and the TEV protease protocol (Sigma, St. Louis, MO, USA) was used to separate the His6 tag anchored to the purified rPirA*^vp^*. For rPirB*^vp^*, the protein was eluted using the same buffer with different concentrations of imidazole, and TRX was removed from the purified protein by incubating with enterokinase (ThermoFisher) at 37 °C overnight. The molecular weights of both purified proteins were verified by SDS-PAGE analysis under reducing conditions using Coomassie blue staining.

### 2.4. Hemagglutinating Activity 

The hemagglutinating activity (HA) of rPirA*^vp^* and rPirB*^vp^* toxins was tested using red blood cells (RBCs) from several animal species and human RBCs from healthy donors. Blood was collected in sterile Alsever’s solution (100 mM glucose, 20 mM NaCl, 30 mM sodium citrate, pH 7.2) and washed three times with phosphate-buffered saline (PBS, 137 mM NaCl, 2.7 mM KCl, 10 mM Na_2_HPO_4_, 1.8 mM KH_2_PO_4_, pH 7.4) by centrifugation (800× *g* for 10 min). The HA was performed in 96-well microtiter U plates (NUNC, Denmark) by a two-fold serial dilution according to the protocol of Fragkiadakis [22]. Briefly, after the two-fold dilution in PBS, 25 μL of each protein’s dilution was mixed with 25 μL of 2% (*w/v*) erythrocytes previously suspended in PBS. The suspension was incubated at room temperature for 120 minutes. The hemagglutinating titer was reported as the inverse of the last dilution exhibiting HA. HA was also assessed in the presence of RBCs treated with sialidase (0.1 U of *Vibrio cholerae* sialidase (Sigma) for every 0.5 mL of packed RBCs at 37 °C for 45 minutes). To determine whether the subunits have protease activity, remazol brilliant blue R (Sigma) was used according to the manufacturer’s instructions. 

### 2.5. Purification of Wild-Type PirAB^vp^ Toxin Complex

The wild-type PirAB*^vp^* toxin complex was purified from the extracellular products (ECPs) of *Vp*M0904 by two affinity chromatography systems. ECPs were obtained by removing the bacterial cells by centrifugation at 4500× *g* for 10 min at 4 °C and filtration at 0.22 µm. The supernatant culture broth was separated and the purification process continued.

Affinity chromatography was done on glutaraldehyde-fixed stroma from Wistar rat’s erythrocytes fixed with glutaraldehyde and immobilized on G-25 Sephadex (Sigma, USA). The rat erythrocytes were fixed following the methodology described by Vazquez et al. [23]. The fixed erythrocytes were packed in a column (1.5 × 25 cm) containing Sephadex G-25 (Pharmacia Fine Chem, Uppsala, Sweden). The supernatant culture broth was dialyzed in potassium/sodium phosphate buffer (KNa PBS, 50 mM NaH_2_PO_4_, 50 mM K_2_HPO_4_, 150 mM NaCl, pH 7.4) and 195.4 mg was loaded onto a column that had been previously equilibrated with the same buffer at room temperature at a flow rate of 15 mL/h. The unbound material was washed from the column with KNa PBS until the absorbance of the eluent was <0.01 at 280 nm. The PirAB*^vp^* toxin was eluted with 3% acetic acid, 1.5 mL fractions were collected, and the pH was immediately neutralized with 1.0 M NaOH. Fractions were pooled and dialyzed with KNa PBS; then, the protein was quantified by the Bradford method [24] and stored at −20 °C until it was used to determine the hemagglutinating activity according to the methodology previously described. 

Affinity chromatography was done on an α-galactose-Sepharose (Invitrogen) column. The α-galactose-sepharose was packed into a column (1 × 5 cm) and equilibrated with sodium/sodium phosphate buffer (NaNa PBS, 50 mM NaH_2_PO_4_, 50 mM Na_2_HPO_4_, 150 mM NaCl, pH 7.4). The remaining supernatant (249 mg) was dialyzed against NaNa PBS and loaded onto the column. The unbound material was washed from the column with NaNa PBS until the absorbance of the eluent was <0.01 at 280 nm. To obtain a fraction containing the PirAB*^vp^* toxin, the column was washed with sodium/sodium phosphate buffer supplemented with 200 mM galactose, collected, dialyzed against KNa PBS, and quantified using the Bradford method [24] before being immediately stored at −20 °C until use to determine the hemagglutinating activity according to the methodology previously described.

HA of wild-type PirA*^vp^* and PirB*^vp^* subunits was not tested because it was not possible to obtain purified subunits with the methodology previously described.

### 2.6. Sugar Specificity and the Requirement of Divalent Cations 

The lectin’s sugar specificity was tested by inhibiting the HA of Wistar rat’s erythrocytes using monosaccharides, oligosaccharides, amino acids, glycosaminoglycans, and glycoproteins. rPirB*^vp^* was diluted in PBS to 2560 HA units (HAU, titer = 64); 25 µL of this dilution was placed on microtiter-U plates and incubated for 30 min at room temperature with 25 µL of each sugar, amino acid, glycosaminoglycan, or glycoprotein at several concentrations in triplicate. Subsequently, 25 µL of 2.0% rat erythrocytes suspension in PBS was added. The following reagents were tested: D-lactose, D-maltose, galactosamine (GalNH_2_), glucosamine (GlcNH_2_), D-galactose, D-glucose, N-acetyl glucosamine (GlcNAc), N-acetyl-galactosamine (GalNAc), D-fucose (D-Fuc), D-mannose (Man), mannitol, xylose (Xyl), saccharose, sorbitol, methyl-glucose (CH3-Glc), methyl-mannose (CH3-Man), melibiose, N-Ac-diacetyl-chitobiose, galacturonic acid, L-arginine (Arg), L-lysine (Lys), L-serine (Ser), cystine, hyaluronic acid, heparin, fetuin (FET), albumin from chicken egg white (OVO), and mucin from the porcine stomach (PSM) (Sigma). The results were expressed as the minimum concentration of carbohydrate, amino acid, glycosaminoglycan, or glycoprotein able to inhibit 2560 HAU. The effect of divalent cations on the HA was assessed by adding several concentrations (from 0.01 to 5 mM) of CaCl_2_ and MgCl_2_. Wild-type and recombinant rPirA*^vp^* did not show hemagglutinating activity against rat erythrocytes, so they were not used in this experiment. 

### 2.7. Production of Polyclonal Antibodies (Pabs) in Rabbit: Anti-rPirA and Anti-rPirB

Two male New Zealand white rabbits (1800 g) were used for this study. Before starting the immunization protocol, each rabbit was dewormed by an oral route with sulfamide-trimethoprim (30 mg/kg/12 h for 10 days), unique doses of ivermectin (0.4 mg/kg), toltrazuril (20 mg/kg), and praziquantel (5 mg/kg). The rabbits were subcutaneously injected along the dorsum at the region of the scapula with a 1.0-mL suspension of recombinant protein (in 50 mM Tris-HCl, pH 8.0) in Freund’s complete adjuvant (Sigma), corresponding to 500 μg/mL of protein, confirmed by the Bradford technique. Four subsequent inoculations of rPirA*^vp^* and rPirB*^vp^* proteins in the same buffer emulsified in Freund’s incomplete adjuvant (FIA) (Sigma) were administered. Rabbits were also subcutaneously injected with 1.0 mL of a suspension of recombinant protein in FIA at the same concentration (500 μg/mL). Blood (3 to 5 mL) was collected from the auricular marginal vein from each rabbit 7 days before the first injection, and then at days 7, 14, 21, and 28 for the measurement of antibody titers. Deep anesthesia was induced with chloroform on day 35, and then blood was withdrawn by cardiac puncture. Blood was incubated at 37 °C for 30 min and was later incubated at 4 °C for 1 h. After the incubation, blood was centrifuged at 6000× *g* for 30 min and serum samples were collected. The polyclonal antibodies (Pabs) were purified by three ammonium sulfate precipitations according to Harlow and Lane [25]; then, they were dialyzed and resuspended in PBS at pH 7.2 and quantified according to Bradford [24]. Samples were stored at −20 °C until use.

### 2.8. Polyclonal Antibodies Specificity 

Antibody (anti-rPirA and anti-rPirB) titration was determined by Western blot, according to Harlow and Lane [25]. Briefly, several concentrations of rPirA*^vp^* and rPirB*^vp^* were separated in 12% SDS-PAGE according to the method described by Laemmli under non-reducing conditions [26]. Samples were electrophoresed for 2 h at 100 V and the gels were electro-blotted onto nitrocellulose membranes using a Transblot apparatus (BioRad Laboratories, Hercules, CA, USA) for 1 h with the transfer buffer (50 mM Tris-HCl, 380 mM glycine, 0.1% SDS, 20% methanol, pH 8.3), according to Towbin [27]. The nitrocellulose membrane was blocked, washed, treated with double serial dilutions of the antibody (1:50 to 1:51,200), and incubated for 1 h at room temperature. After extensive washing in PBS-Tween 20, the membrane was incubated with anti-rabbit-HRP (Invitrogen) at 1:3000 for 1 h. To reveal activity, the membrane was extensively washed and incubated for 5 min in a substrate solution containing 0.02% diaminobenzidine (DAB), 0.03% hydrogen peroxide and 0.015% cobalt chloride in PBS-Tween 20. The antibody titer was the value of the highest dilution in which the proteins were observed in the membrane.

### 2.9. Immunodetection of PirA^vp^ and PirB^vp^

To detect the presence of PirA*^vp^* and PirB*^vp^* proteins of *Vp*M0904 in both ECPs and the fractions of purification, the anti-rPirA’s and anti-rPirB’s Pabs were used in the Western-blot assay. After 12% SDS-PAGE under non-reducing conditions, the gels were blotted on a nitrocellulose membrane, incubated in blocking buffer, and probed with the appropriate primary antibody for 1 h at room temperature (1:12,000 for PirA*^vp^* and 1:300 for PirB*^vp^*). After washing 3 times with PBS-Tween 20, the membrane was incubated with the secondary antibody, horseradish peroxidase-conjugated F(ab’)_2_ fragment of anti-rabbit IgG (Sigma), for 1 h at room temperature. A spot was revealed after washing with PBS-Tween 20 using DAB and hydrogen peroxide in the same buffer. For the identification of wild-type PirA*^vp^*, it was necessary to perform the complete procedure in the presence of 200 mM galactose. Some samples were electrophorezed for two h at 100 V and the proteins were visualized in the gel using the silver staining protocol [28]. 

## 3. Results

### 3.1. Purification of Recombinant Proteins

**Figure 1:**
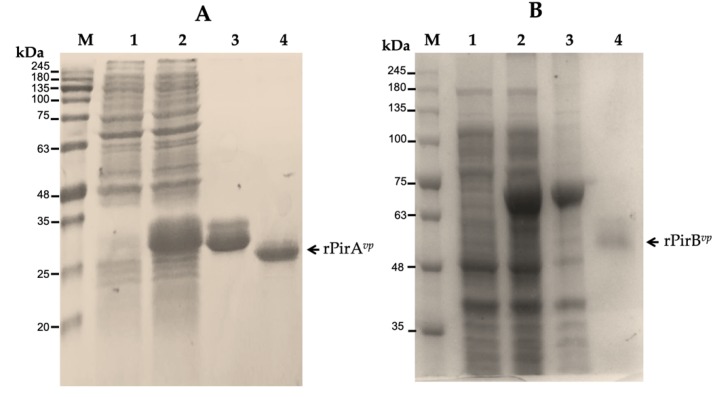
SDS-PAGE analysis of rPirA*^vp^* and rPirB*^vp^* toxins of *V. parahaemolyticus*. (**A**) M, molecular maker; lane 1, lysate of *E. coli* non-induced; lane 2, lysate of induced culture after 16 h; lane 3, rPirA*^vp^* purified with 500 mM imidazole; lane 4, rPirA*^vp^* after TEV protease treatment. (**B**) M, molecular maker; lane 1, lysate of *E. coli* non-induced; lane 2, lysate of culture after 4 h; lane 3, rPirB*^vp^* purified with 300 mM imidazole; lane 4, rPirB*^vp^* after enterokinase treatment.

Both recombinant proteins were obtained in *E. coli* lysates at different incubation times and purified by Ni-affinity. The PirA*^vp^* recombinant that was eluted using 500 mM imidazole appeared on the SDS-PAGE as a band with an apparent molecular weight of about 25 kDa, probably the dimeric nature of this protein (Figure 1A). The PirB*^vp^* recombinant protein was eluted with 300 mM imidazole, and it was identified as a single band with an apparent molecular weight of about 66 kDa because it was co-expressed with a thioredoxin tag to promote solubility. Treatment with enterokinase produced a band with an apparent molecular weight of 50 kDa (Figure 1B). The protein profile of lysates of *E. coli* showed differences between induced and non-induced cells. The molecular weights for both proteins were consistent with those calculated as reported previously: 50.1 kDa for PirB*^vp^* and 12.7 for PirA*^vp^*.

### 3.2. HA of the rPirB^vp^ and Wild-Type PirAB^vp^ Toxin

The rPirB*^vp^* purified by Ni-affinity was capable of agglutinating erythrocytes from rats (Wistar strain) but failed to agglutinate red cells from rabbits; A, B, and O human types; ovine species; and mouse (BalbC strain). rPirA*^vp^* did not show HA against any of the erythrocytes tested. Likewise, the wild-type PirAB*^vp^*, purified from ECPs with stroma and α-galactose column, agglutinated only rat erythrocytes. When the rat erythrocytes were desialylated, there was a 4.0-fold increase in the agglutinating activity of rPirB*^vp^* (Table 1). 

Native PirA*^vp^* and PirB*^vp^* subunits could not be purified. In addition, rPirA*^vp^* and rPirB*^vp^* did not have proteolytic activity, confirmed by the assay with remazol brilliant blue R.

### 3.3. Purification of Wild-Type PirAB^vp^ Toxin Complex 

**Figure 2:**
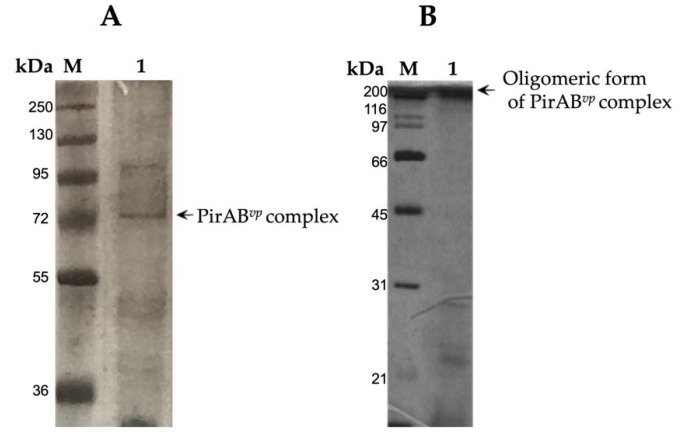
SDS-PAGE of the PirAB*^vp^* toxin purified by affinity chromatography. M, molecular marker. (**A**) Lane 1, fraction obtained from a column with rat stroma of extracellular products (ECPs) eluted with 3% acetic acid. (**B**) Lane 1, fraction purified from ECPs onto α-galactose column eluted with 200 mM galactose.

The PirAB*^vp^* from ECPs of *Vp*M0904 was identified and purified in a single step by affinity chromatography on glutaraldehyde-fixed stroma from rat erythrocytes (Figure 2A) or α-galactose-Sepharose (Figure 2B). The purified complex toxin with stroma had an apparent molecular weight of 70 kDa, which was not observed in the α-galactose affinity purification. The procedure using α-galactose-sepharose showed a 250 kDa band, which apparently corresponded to the tetrameric complex formed of four subunits of PirA*^vp^* (12 kDa for each one) and four subunits of PirB*^vp^*(50 kDa for each one).

In the purification of the PirAB*^vp^* using a rat stroma column, a 199-fold increase in the specific activity of the toxin complex was observed with this procedure compared with the ECPs (Table 2). The concentration of the toxin eluted from the column represented 0.50% of the total protein present in ECPs. 

In the purification of the PirAB*^vp^* toxin in the galactose column, a 174-fold increase in the specific activity of the PirAB*^vp^* toxin was observed with this procedure compared with the ECPs (Table 3). The concentration of PirAB*^vp^* toxin eluted from the column represented 0.57% of the total protein present in ECPs. Also, there were differences in hemagglutinating activity units of the PirAB*^vp^* purified by the two affinity chromatographic methods, 160 (titer = 4) for the eluted rat stroma complex and 640 (titer = 16) for the galactose purified complex. 

### 3.4. Sugar Specificity and the Effect of Divalent Cations on the HA of rPirB^vp^

GalNH_2_ and GlcNH_2_ were better sugar inhibitors for rPirB*^vp^* HA; 25 mM was the minimal concentration to inhibit 2560 HAU of rPirB*^vp^*, whereas, 4-fold higher concentrations of the disaccharides, Mal and Lac, were necessary to inhibit a similar activity of the rPirB*^vp^*. The amino acid, Arg, and the cysteine dimer, cystine, present the same activity as the amino-sugars (Table 4). Other carbohydrates, such as Gal, Glc, Man, GlcNAc, GalNAc, Fuc, mannitol, xylose, sorbitol, Met-glucopyranoside, Met-mannose, melibiose, N,N’-Ac-diacetyl-chitobiose, sialic acid, sucrose, and the amino acids, Lys and Ser, had no inhibitory activity at 200 mM. Heparin showed a relative inhibitory potency, 93-fold higher compared with the amino-sugars tested, whereas hyaluronic acid had no inhibitory activity. The FET glycoprotein showed the strongest inhibition for rPirB*^vp^*, whereas OVO showed a lower inhibition, and PSM showed no effect on the rPirB*^vp^* HA. No effect of divalent cations, such as Ca^2+^ and Mg^2+^, was observed on PirB*^vp^* lectin hemagglutinating activity at a concentration of 5 mM (data not shown).

### 3.5. Specificity of Polyclonal Antibodies

Each protein was used for the production of polyclonal antibodies in rabbits, and they were used to confirm the presence of proteins during the purification procedure. To determine the minimum dilution for the detection of rPirA*^vp^* or rPirB*^vp^*, 1 μg of total protein was run on a single-well gel, electro-blotted, and detected by serial dilutions of 1:50 to 1:51, 200 (0.093 to 96 μg of antibody); 0.9 and 16 μg were the concentrations of anti-rPirA and anti-rPirB antibody that were necessary to detect each protein (1:12,000 and 1:300, respectively).

**Figure 3:**
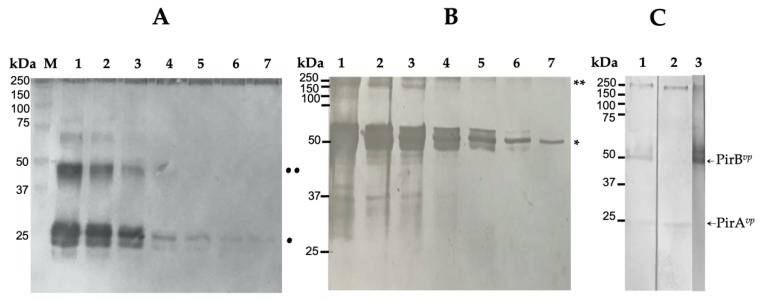
Specificity and sensitivity of polyclonal antibodies. Minimum detectable concentration of rPirA*^vp^* (**A**) and rPirB*^vp^* (**B**) using anti-rPirA (1:12,000) and anti-PirB (1:300) antibodies. (**A**) M, molecular marker; lanes 1–7: 1260, 632, 316, 158, 79, 39, and 19.5 ng of rPirA*^vp^*; (•) dimeric form; (••) tetrameric form. (**B**) lanes 1–7: 2940, 1470, 735, 368, 184, 92, and 46 ng of rPirB*^vp^*; (*) monomer; (**) tetrameric or pentameric form. (**C**) antibodies specificity against ECPs from *Vp*M0904 tested with anti-rPirA. Lane 1 and 2, free and plus 200 mM galactose, respectively; lane 3, anti-rPirB antibody.

Both recombinant proteins were identified by SDS-PAGE using reducing conditions of each fraction obtained with different concentrations of imidazole (Figure 1). rPirA*^vp^* was identified in the dimeric (24 kDa) and tetrameric (48 kDa) forms, the dimeric form was the most abundant (Figure 3A) in non-reducing conditions. rPirB*^vp^* was identified as a 50 kDa monomer and probably as a 200–250 kDa tetra- or pentameric form under the same conditions (Figure 3B).

Sensitivity of the anti-rPirB Pabs versus rPirB*^vp^* was approximately 46 ng of the recombinant protein per band (Figure 3B, lane 7); the anti-rPirA Pabs specific for rPirA*^vp^* had a sensitivity that corresponded to approximately 19.5 ng of protein per spot (Figure 3A, lane 7).

Similarly, each subunit of the native protein was recognized by the antibodies obtained in this work. Anti-rPirA Pabs recognized a 24 kDa fraction and anti-rPirB Pabs detected a 50 kDa band (Figure 3C, lane 1). Anti-rPirA showed cross-reaction with a 50 kDa protein, which was eliminated using 200 mM galactose (Figure 3C, lane 2), and anti-rPirB*^vp^* only detected a 50 kDa band in the ECPs of *Vp*M0904 (Figure 3C, lane 3).

## 4. Discussion

Bacterial lectins play a key role in eliminating phylogenetically-related antagonists for the adhesion or colonization of specific niches, such as tissues of hosts or for biofilm formation. This implies the need for the secretion of toxins with lectin activity that allows oligo- and polysaccharides to be recognized in the targets. In addition, lectins can act as recognition molecules in cell–molecule and cell–cell interactions in a variety of biological systems [29,30]. The first reports of bacterial lectins were from *Pseudomonas*; their toxins have been associated with virulence factors [31,32,33,34]. *P. aeruginosa* produces a variety of toxins with lectin-like activity that are known as bacteriocins and pyocins that agglutinate bacteria to inhibit the growth of competitors [31,32]. This kind of lectin has been characterized in *P. putida*, *P. fluorescens*, *P. syringae*, and *Xanthomonas citri* [34,35,36,37,38,39]. The Tor biotype of *V. cholerae* expresses the lectin-like toxin called cell-associated mannose-sensitive hemagglutinin (MSHA), which is a putative attachment factor [40,41,42]. 

The results from the present study suggest that the PirB*^vp^* subunit of the PirAB*^vp^* binary toxin secreted by the marine bacterium, *V. parahaemolyticus*, the causal agent of AHPND in shrimp, has lectin activity. The PirB*^vp^* subunit binds to a molecule containing amino sugars, such as glycosaminoglycans linked to proteoglycans that could have a receptor function on the membrane of target cells in shrimp tissues. 

Although the wild-type PirAB*^vp^* protein can be purified using two procedures, the amounts obtained of the total protein secreted by *Vp*M0904 was very low in both cases: 0.50% using a stroma column and 0.57% with a galactose column.

In the present study, *E. coli* cells were successfully transformed. Cells produced 6000-times more recombinant PirA*^vp^* and PirB*^vp^* than *Vp*M0904 cells can produce. This quantity was enough to determine the hemagglutinating activity and specificity for sugars of both proteins, as reported in previous studies on characterizations of other *Vp* proteins [8,43,44]. The PirB*^vp^* subunit recognized only rat erythrocytes, which contain large amounts of 9-*O*-acetyl sialic acid (Neu5,9Ac), above 25–30% of sugars in their cell membranes and other *O*-glycoconjugates with galactose or galactosamine residues [45,46]. The former sugar could bind to the PirB*^vp^* subunit and recognize the amino group; however, elimination of the sialic acid residues (Neu5,9Ac) from rat erythrocytes showed an increase in the hemagglutination activity. This result demonstrated that the potential negative charge of the sialic acid was not as decisive for the PirAB*^vp^* toxin–ligand interactions, such as already been reported for other lectins [47]. Likewise, this indicates that the carbohydrate determinants on these red blood cells are partially accessible, and after partial removal of the sialic acid from the erythrocytes’ surface, they became more accessible. We propose that the lectin activity is specific for glycoconjugates containing N-acetylglucosamine or N-acetylgalactosamine in a terminal non-reducing position [48]. Therefore, the results suggest that the recognition of the receptor is not sialic acid-dependent.

During the purification process of proteins using rat stroma, a complex containing PirA*^vp^* and PirB*^vp^* subunits was formed. The complex was purified as an oligomer of four PirA*^vp^* subunits and four PirB*^vp^* subunits when using galactose. Probably, these differences between the structures obtained by each purification process were due to the recognition of the complex of different molecules. Thus, whereas the stroma column has several sugar sequences, the galactose column has only one, that could facilitate the purification of a tetrameric complex by the oligomerization, induced by recognition of the same sugar, as previously reported for the lectin purified from *Mytilus californianus* [49]. Also, the use of acetic acid in a stroma column to dialysis the protein did not allow a reversion to the same quaternary structure that was previously eluted. This did happen in the column of galactose, which was eluted with the same sugar. For this reason, a band of approximately 70 kDa molecular weight was observed, probably formed by one PirA*^vp^* plus one PirB*^vp^* subunit. Evidently, the PirAB*^vp^* toxin belongs to AB binary toxins composed of one A subunit of 12 kDa and one B subunit of 50 kDa. 

We found that the hemagglutinating activity of the PirB*^vp^* subunit is higher if it is attached to PirA*^vp^*. Furthermore, PirA*^vp^* alone did not show activity against any type of erythrocytes tested. Possibly the A subunit participates in stabilizing the complex for a better binding to the possible receptor molecule on the shrimp hepatopancreatic epithelial cells [50]. These results highlight the importance of the interaction between the two subunits of the complex, which could act in molecular synergy to recognize the target molecule, as has been reported for the A and B subunits of the *R. communis* toxin [51]. In agreement with the above, Lin et al. [52] suggested that the PirA*^vp^* subunit probably acts to give structural stability to the PirAB*^vp^* toxin. Our results contribute to the understanding of the structural characterization of the PirAB*^vp^* toxin and, in turn, the type and chemical structure of the saccharides present in possible receptor molecules. Most authors suggest that the PirAB*^vp^* binary toxin acts as a pore-forming toxin, killing epithelial cells that induce AHPND in shrimp. In silico analysis based on the structural similarity between PirAB*^vp^* and *Bacillus thuringiensis* Cry toxins support this theory [50,53]. However, this has not been experimentally demonstrated yet. The data presented here demonstrate that the PirB*^vp^* toxin has lectin activity, which is possibly involved in the recognition of a receptor molecule. 

The inhibition with amino sugars suggests that the possible receptor or adhesion molecule can be a proteoglycan or glycolipid of the epithelial cells of the shrimp hepatopancreas [54]. The PirB*^vp^* lectin activity was inhibited by GlcNH_2_, GalNH_2_, lactose (Galβ1,4Glc), maltose (Glcα1,4Glc), arginine, cystine, heparin, OVO, and FET. The fact that lactose (Galβ1,4Glc) and maltose (Glcα1,4Glc) inhibit the hemagglutinating activity of PirB*^vp^* at the same concentration reveals that the recognition can be based on the glycosidic bond coupled with one of the monosaccharides that form the sugar. Probably, the PirB*^vp^* subunit may recognize the α glycosidic bond between two sugar units, suggesting that the sequence of the Gal(β1-3 or 4)GlcNAc(α 1-2)Man sugar is essential for the PirB*^vp^* subunit interaction with the host structures. The existence of heparan sulfate (HS) chains was reported in shrimp [55]. These are sulfated glycosaminoglycans (GAGs) in syndecans involved in several steps of infection caused by the white spot syndrome virus, including initial attachment and entry of viral proteins in shrimp. Besides, the inhibition in the presence of the amino acids, arginine and cysteine, suggest that a negative charge in the membrane of the erythrocyte can mediate interactions with the positive charge of the arginine residue [56]. Positive residues, like arginine, lysine, and histidine, usually participate in hydrogen bonding with some receptors and the positive charge can enhance the complex stability in protein–protein interactions [57,58]. 

Heparin is another inhibitor of the hemagglutination activity, and its inhibition was 93-times higher than that of amino acids or amino-sugars. The GAGs, such as heparin/HS, are synthesized and serine-linked to the core proteins of proteoglycans, and they are located on the plasma membrane and in the extracellular matrix [59]. These data reveal a PirB*^vp^–*GAGs interaction mediated by a sequence of sugars of heparin or HS binds to a proteoglycan of the epithelial cells. The effects of these GAGs are exerted through their capacity to engage protein ligands [60]. Many pathogens produced proteins that interact with heparin/HS as part of their molecular adaptation to infection of mammals [61]. Probably, the B subunit may recognize a sequence with *O*-amino sugars, stabilized by the negative charge of the sulfate groups possessed by these GAGs. Also, the presence of numerous negatively charged sulfate groups in GAGs provides an ideal multi-landing pad for proteins and macromolecules through electrostatic interactions [62]. 

The most powerful glycoprotein inhibitor is fetuin, which contains *O*-glycosidically-linked oligosaccharide chains [63]. However, ovalbumin, which was also inhibitory, contains a single *N*-glycosdically-linked glycan chain at Asn-392 [64]. Interestingly, the glycoproteins with inhibitory capacity possess as predominant oligosaccharides either the sequence NeuAc(α2,6)Gal(β1,3)GalNAc(αl,*O*)Ser/Thr or NeuAC(α2,3)GalNAc(α1,*O*)Ser/Thr [65], which may explain why both proteins have an inhibitory capacity. Mucin from porcine stomach, a glycoprotein containing *O*-glycosdically-linked glycan chains (Galβ1,3GalNAc) [66], did not inhibit the hemagglutinating activity of PirB*^vp^*. Possibly, this is due to PirB*^vp^* recognizing the difference between the α1-*O* bond of Ser/Thr and the β1-*O* bond present in mucin. The predominant component of bovine submaxillary mucin is 9-*O*-acetyl- and 8,9-di-*O*-acetyl-*N*-acetyl-NeuAC [47], which explains why this sequence is not recognized by the B subunit. The above suggests that the PirB*^vp^* subunit contains a binding site for glycans in the binding site of a possible receptor on the epithelial cells of the shrimp hepatopancreas. Inhibition of the lectin activity of the PirB*^vp^* subunit was observed in the presence of Ca^2+^ and Mg^2+^ ions; possibly, the ions caused structural changes in the subunit, which resulted in the loss of activity [67]. Data of this study contribute to the knowledge of the interactions of the PirB*^vp^* subunit with the receptor. Although the principal interaction can be with glycosaminoglycans in the receptor structure, a positive charge is necessary for protein–protein interactions to have more stability and produce damage in the hepatopancreas of shrimp.

## 5. Conclusions

The PirB*^vp^* subunit integrated into a tetrameric PirAB*^vp^* complex can recognize glycosaminoglycans molecules (amino sugars) and probably binds to the receptor molecules on the membrane of the hepatopancreatic epithelial cells of shrimp to trigger the massive sloughing of these cells. However, the specific role of the PirB*^vp^* subunit as a lectin, as well as the function of the PirA*^vp^* subunit, in the pathogenesis of AHPND have not yet been determined. 

## Figures and Tables

**Table 1:** Hemagglutination activity assay of the recombinant and wild-type toxins. The tests were performed in the presence of 2% erythrocyte suspension in PBS, pH 7.4.

Red Blood Cells	Hemagglutinating Activity (HA) ^a^		
	rPirA*^vp^*	rPirB*^vp^*	PirAB^vp^*
Human A	-	-	-
Human B	-	-	-
Human O	-	-	-
Rabbit	-	-	-
Mouse (BalbC)	-	-	-
Rat (Wistar)	-	81,476	114,285
Desialylated Rat ^b^	-	325,970	ND
Ovine	-	-	-

rPirA*^vp^* was 1.12 μg/25 μL; rPirB*^vp^* was 0.8 μg/25 μL; PirAB*^vp^* was 0.035 μg/25 μL. ^a^ HA: hemagglutinating activity is reported as the inverse of the last dilution showing visible agglutinating activity. ^b^ Erythrocytes of rat were incubated in presence of *Vibrio cholera* sialidase 45 min at 37 °C using rPirB*^vp^* for the activity assay. PirAB^vp^* purified using α-galactose column. -: No hemagglutinating activity. ND: not determined hemagglutinating activity.

**Table 2:** Purification process of the PirAB*^vp^* toxin from ECPs of *Vibrio parahaemolyticus* in rat stroma.

Fraction	Total Protein (mg)	HAU ^a^	Specific Activity ^b^
ECP	195.4	160.0	0.82
Unretained fraction	194.42	0.0	0.0
Retained fraction (PirAB*^vp^*)	0.980	160.0	163.26

^a^ HAU = hemagglutinating activity units tested in presence of rat erythrocytes. ^b^ Specific activity = HAU/mg protein.

**Table 3:** Purification process of the PirAB*^vp^* toxin of ECPs from *Vibrio parahaemolyticus* in α-galactose.

Fraction	Total Protein (mg)	HAU ^a^	Specific Activity ^b^
ECP	249.24	640.0	2.56
Unretained fraction	247.80	0.0	0.0
Retained fraction (PirAB*^vp^*)	1.43	640.0	447.55

^a^ HAU = hemagglutinating activity units tested in presence of rat erythrocytes. ^b^ Specific activity = HAU/mg protein.

**Table 4:** Specificity of PirB*^vp^* subunit for carbohydrates and glycoproteins.

Inhibitor	Concentration (mM) ^a^	Relative Inhibitory Potency ^b^
Lac	100	1
Mal	100	1
GalNH_2_	25	4
GlcNH_2_	25	4
Arginine	25	4
Cystine	25	4
Heparin	0.27	370.4
OVO	0.015	6666.7
FET	0.00031	322,580.6

^a^ Minimum concentration to inhibit 2560 hemagglutinating activity units (HAU) of the rPirB*^vp^* lectin in presence of 2% rat erythrocytes suspension in PBS, pH 7.4. ^b^ Relative inhibition capacity compared with lactose or maltose. Other carbohydrates without inhibitory capacity, even at 200 mM, were GalNAc, GlcNAc, Glc, Gal, Man, GlcNAc, GalNAc, Fuc, mannitol, xyl, sorbitol, CH3-glucopyranoside, CH3-mannose, melibiose, N,N’-Ac-diacetyl-chitobiose, sialic acid, sucrose, and amino acids (Lys and Ser), hyaluronic acid; glycoproteins without inhibitory capacity were mucin from porcine stomach and bovine submaxillary mucin.
